# Tumor-associated macrophages: new insights on their metabolic regulation and their influence in cancer immunotherapy

**DOI:** 10.3389/fimmu.2023.1157291

**Published:** 2023-06-22

**Authors:** Li Xiao, Qiao Wang, Hongling Peng

**Affiliations:** ^1^ Department of Obstetrics and Gynecology, West China Second Hospital, Sichuan University, Chengdu, Sichuan, China; ^2^ Development and Related Diseases of Women and Children Key Laboratory of Sichuan Province, Key Laboratory of Birth Defects and Related Diseases of Women and Children, Ministry of Education, West China Second University Hospital, Sichuan University, Chengdu, Sichuan, China

**Keywords:** tumor-associated macrophages, glucose metabolism, amino acid metabolism, fatty acid metabolism, immunotherapy, tumor microenvironment

## Abstract

Tumor-associated macrophages (TAMs) are a dynamic and heterogeneous cell population of the tumor microenvironment (TME) that plays an essential role in tumor formation and progression. Cancer cells have a high metabolic demand for their rapid proliferation, survival, and progression. A comprehensive interpretation of pro-tumoral and antitumoral metabolic changes in TAMs is crucial for comprehending immune evasion mechanisms in cancer. The metabolic reprogramming of TAMs is a novel method for enhancing their antitumor effects. In this review, we provide an overview of the recent research on metabolic alterations of TAMs caused by TME, focusing primarily on glucose, amino acid, and fatty acid metabolism. In addition, this review discusses antitumor immunotherapies that influence the activity of TAMs by limiting their recruitment, triggering their depletion, and re-educate them, as well as metabolic profiles leading to an antitumoral phenotype. We highlighted the metabolic modulational roles of TAMs and their potential to enhance immunotherapy for cancer.

## Introduction

Tumor microenvironment (TME) has received increasing attention in recent years, due to its critical role in tumor immune suppression, distant metastasis, local resistance, and target therapy response. TME is regulated by crosstalk within and between all of its cellular components, including tumor-associated macrophages (TAMs). The ‘immunosuppressive and protumoral’ behavior of TAMs, according to emerging evidence, is caused by a rewired metabolic program that influences cancer disease progression and outcome.

At the stage of tumor initiation, TAMs, a major component in TME, stimulate antitumor immunity. The bulk of TAMs has been recruited locally from bone marrow-derived peripheral blood monocytes in response to chemokines and growth factors (1). Similar to other immune cells, TAMs acquire a broad range of phenotypic and functional states in response to environmental signals. Macrophages are capable of polarization into two forms with distinct functions: classically activated macrophages (M1) and alternatively activated macrophages (M2) (2). Interferon (IFN)-γ induces the typically activated M1 macrophages, whereas Interleukin (IL)-10, IL-4, and IL-13 produce the alternatively activated M2 macrophages (3). TAMs are typically M2-like macrophages, which express higher quantities of anti-inflammatory cytokines, scavenger receptors, angiogenic factors, and proteases than M1-type macrophages (1). Transcriptome analysis reveals, however, that TAMs express a mixture of both M1 and M2 genes, rather than demonstrating a unique M1 or M2 phenotype (4). Based on single-cell mRNA sequencing, researchers have categorized TAMs activation types beyond the conventional M1/M2 polarization model, revealing that there are continuous intermediate phenotypes with different roles and functional states (5). A macrophage polarization spectrum model has been recommended, which better illustrates a large variety of macrophage responses to stimuli (6). TAMs are phenotypically and functionally heterogeneous; hence, some can promote tumor growth while others can exert antitumor action (7).

The major function of macrophages is to identify and phagocytose antigens, then present them to T cells. By producing mediators such as IL-1β, IL-6, tumor necrosis factor (TNF)-α, C-C motif chemokine (CCL)2, C-X-C motif chemokine (CXCL)8, CXCL10, vascular endothelial growth factor (VEGF), platelet-derived growth factor (PDGF), transforming growth factor (TGF)-β and fibroblast growth factor (FGF), TAMs can promote tumor development and remodel the tumor-supportive TME (8–10). There is substantial clinical and experimental evidence that TAMs are pivotal orchestrators of cancer-related inflammation because they promote tumor initiation, and metabolic alterations, stimulate tumor angiogenesis, enhance tumor cell migration, and progression to malignancy, and suppress antitumor immunity (6, 11, 12). Hence, there is evidence that in developing tumors, TAMs exhibit an M1-like phenotype and can remove immunogenic tumor cells. Subsequently, tumor progression is accompanied with skewing and subversion of macrophage function by stimuli within TME that could induce a protumorigenic M2-like polarization of TAMs (11, 13).

TAMs exhibit a high degree of functional flexibility and a modified metabolism, which is exemplified by their heightened sensitivity to TME. Increasing evidence suggests that the metabolic characteristic of TME affects the differentiation, mobilization, polarization, and antitumor immune responses of TAMs. For classical macrophage polarization, M1-like macrophages preferentially are connected with a highly glycolytic metabolism, whereas alternatively, activated M2-like macrophages derive the majority of their energy from fatty acid (FA) oxidation (14). Oxidative phosphorylation (OXPHOS), reduced glycolysis and pentose phosphate pathway (PPP) and fatty acid oxidation (FAO), and increased arginase characterize the metabolism of IL-4/IL-13-activated macrophages (15). The metabolism of TAMs offers novel therapeutic possibilities for the treatment of cancer. However, neither the metabolic profiles nor the mechanisms underlying TAMs metabolism are well understood. Here, we assess the interplay between the metabolism and functional reprogramming of TAMs, as well as promising approaches that target TAM metabolic modulation to mitigate the immunosuppressive nature of the tumor microenvironment, thereby enhancing the effectiveness of cancer immunotherapies.

## Metabolic alterations in TAMs

**Figure 1:**
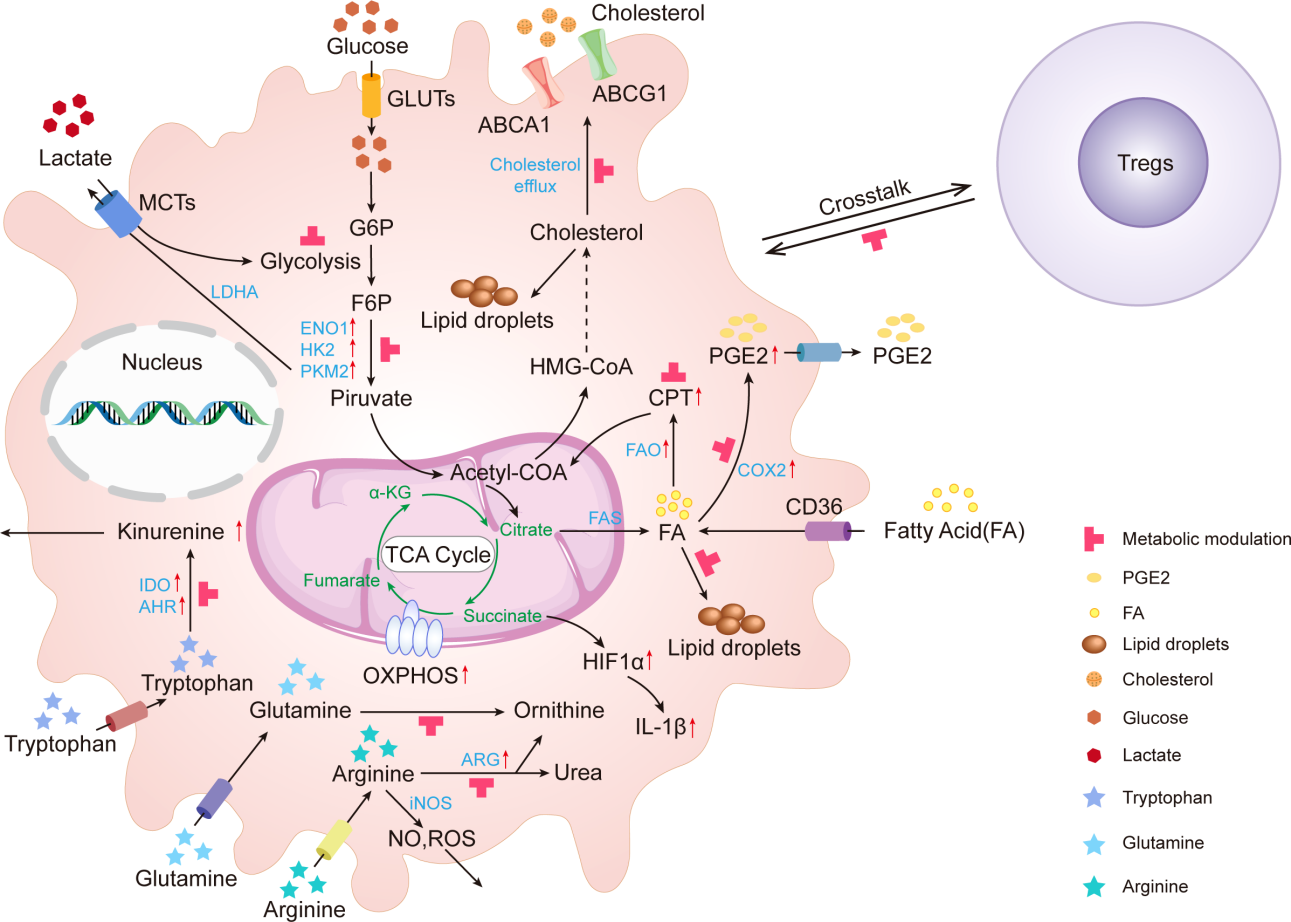
Metabolic alterations in tumor-associated macrophages. In TME, glucose, amino acid, and lipid metabolites activate TAMs. These activated TAMs consume glucose, lactate, tryptophan, arginine, glutamine, fatty acid, and cholesterol leading to the depletion of these metabolites. Upward red arrows indicate upregulation. The red T-shaped symbol indicates the metabolic modulating targets. IDO, Indoleamine 2,3-Dioxygenase; GLUT, glucose transporter; MCTs, monocarboxylate transporters; HK2, Hexokinase II; ENO, enolase; LDHA, lactic acid dehydrogenase; PKM2, Pyruvate Kinase M2; ROS, Reactive Oxygen Species; ARG, Arginase; NO, Nitric oxide; HIF, Hypoxia inducible factor; TCA cycle, Tricarboxylic Acid Cycle; α-KG, α-ketoglutarate; PGE2, Prostaglandin E_2_;; FA, Fatty acid; COX-2, Cyclooxygenase-2; CPT1, Carnitine Palmitoyltransferase 1; OXPHOS, Oxidative Phosphorylation; AHR, Aryl Hydrocarbon Receptor; FAO, Fatty Acid Oxidation; FAS, Fatty Acid Synthesis; ABCG1, ATP-binding Cassette Transporter G1; ABCA1, ATP binding cassette transporter A1; G6P, Glucose-6-phosphate; F6P, Fructose-6-phosphate.

Accumulating evidence suggests that TAMs metabolism is crucial to the signaling network that regulates the expression of specific transcriptional programs. Metabolic adaptation is a crucial characteristic of macrophage plasticity and polarization. M1 and M2- polarized macrophages display a distinctive metabolic profile involving iron, amino acids (AA), glucose, and lipids, all of which have a substantial effect on their immune functions (16). As the metabolic program of TAMs can regulate their pro-tumoral functions, there is strong interest in elucidating the cellular pathways underlying the TAMs’ phenotype. TAM differentiation, mobilization, polarization, and antitumor immune responses, conversely, are regulated by the metabolism in the TME. However, the metabolic profile of TAMs is extremely dynamic, fluctuating in response to adjustments in TME and the nutritional requirements of tumor cells (**Figure 1**).

### Glucose metabolism In TAMs

TAMs prioritize glycolysis as a major metabolic pathway, which sets them apart from the typical M2-polarized subgroup physiologically. Citrate that is produced during glucose oxidation can be converted into acetyl-CoA (Ac-CoA), the precursor for fatty acid (FA) synthesis. Glycolysis is interconnected to PPP via intermediate glucose-6-phosphate(G6P), amino acid metabolism via intermediate 3-phosphoglycerate, and FA metabolism via pyruvate into the tricarboxylic acid (TCA) cycle (16). The process known as “Warburg metabolism” occurs when cancer cells use aerobic glycolysis to meet their energy needs even when there is oxygen present. Even under normal conditions, cancer cells can exhibit the Warburg effect, which is a phenomenon in which they switch their energy metabolism toward glycolysis (14). The elevated glycolysis allows macrophages to respond to intruding pathogens in terms of proinflammatory cytokine production, enhances phagocytosis capacity, and generates sufficient Adenosine triphosphate (ATP) and biosynthetic intermediates to perform their specific effector functions (6, 17). Emerging studies have shown the upregulation of enzymes hexokinase-2 (HK2), phosphofructokinase, enolase 1 (ENO1), and pyruvate kinase M2 (PKM2) in animal models and human cells stimulated with tumor extract solution (18, 19). The ability of TAMs to function on relatively modest nutritional inputs as found in TME is supported by the fact that they display lower glyceraldehyde 3-phosphate dehydrogenase (GAPDH) and succinate dehydrogenase (SDH) activity than normal macrophages (20). Glucose metabolism within tumor cells accentuates anaerobic glycolysis and diminishes oxidative phosphorylation, resulting in an increase in lactate production (21).

On the one hand, the consumption of large quantities of glucose by tumor cells is likely to inhibit glycolysis in TAMs, thereby restraining their antitumor-effect functions. TAMs, on the other hand, can switch from oxidative phosphorylation to glycolysis in response to cancer cell-derived signals, and sustained glycolysis generates copious amounts of lactate. Lactic acid is transported across biological membranes through reversible monocarboxylate transporters (MCTs). The most ubiquitously expressed family member, MCT1, facilitates lactate and pyruvate exchange. Notably, MCT1 inhibition increased the levels of glucose- and fructose-6-phosphate, fructose-bisphosphate, and glycerol-3-phosphate, and markedly reduced products of the ATP-generating arm of glycolysis (22). Lactate, a dead-end waste product of glycolysis, plays a key role in regulating tumor immune surveillance and immune function. Through the production of immunosuppressive cytokines, Lactate is also known to promote M2-like polarization of TAMs that promote tumor progression, angiogenesis, and epithelial-mesenchymal transition (EMT) (23). Lactate promotes angiogenic response and M2-like macrophage accumulation. The impact of lactate on M2 macrophage polarization is diminished when lactate synthesis in cancer is suppressed (24). Elevated lactic acid concentration triggers HIF-1α and mTOR-dependent aerobic glycolysis (25), increases VEGF secretion mediated by MCTs (26), and stimulates CCL5 secretion through Notch signaling in macrophages (27). Through encouraging the production of lactic acid dehydrogenase (LDHA) and pyruvate dehydrogenase kinase (PDK), HIF-1α is a crucial determinant of how cells convert pyruvate into lactate (28). Overexpression of HIF-1α stimulates glycolysis and PPP intermediates in macrophage, which induced M1 macrophage polarization (29). In an acidic tumor environment, G protein-coupled receptor 132 (Gpr132) functions as a key macrophage sensor of the increased lactate. Lactate activates Gpr132 to promote alternatively activated macrophage (M2)-like phenotype, uncovering the lactate-Gpr132 axis as a driver of breast cancer metastasis by stimulating tumor-macrophage interaction (30).

### Amino acid metabolism in TAMs d

Amino acid switching is an important metabolic characteristic and has a fundamental influence on TAM phenotypic polarization. TAMs with amino acid-restricted have a phenotype that is antitumoral, showing decreased TAM infiltration, tumor development, and improved immunotherapy response (31).

Plasma glutamine is the most abundant amino acid and a key intermediate in the tricarboxylic acid (TCA) cycle. Glutaminolysis is essential for the alternative activation of M2-like macrophages, which is accompanied by FA oxidation (FAO) and JMJF3-dependent epigenetic reprogramming of M2 genes (32). Numerous prokaryotic and eukaryotic species utilize glutamine synthetase (GS), a glutamate-metabolizing enzyme, for nitrogen metabolism, acid-base balance, and cell signaling (33). A high correlation exists between GS enzyme activity and TAM polarization to immunosuppressive M2-like TAMs (34). Numerous components of TAMs are regulated by glutamine catabolism, and it has been postulated that glutamine catabolism plays an important role in immunological function in the context of cancer. In malignancies of different cancer subtypes, glutaminase (GLS) transcripts are elevated. Deprivation of glutamine or suppression of N-glycosylation diminished M2 polarization and chemokine CCL2 production. Aspartate-aminotransferase, a key shunt enzyme, consistently reduced the generation of nitric oxide and interleukin-6 in M1 macrophages (35). Overall, the synthesis of glutamine by GS renders a protumoral phenotype in TAMs. The fundamental mechanism by which glutamine metabolism facilitates macrophage activation and elicits desirable immune responses remains unknown.

The involvement of arginine metabolism in nitric oxide (NO) generation or arginase pathway in defining M1- and M2-like macrophage polarization is essential (36). M1 macrophages produce iNOS, which converts arginine to NO and L-citrulline. M2 macrophages contain significant quantities of arginase 1 (ARG1), which converts arginine to ornithine and urea (37). The gene expression profile of TAMs isolated from murine fibrosarcoma demonstrates an elevated pattern of immunosuppressive gene expression and a low level of iNOS expression (38). *In vitro*, TAMs that overexpress ARG1 has a growth-promoting effect on breast cancer cells due to enhanced ARG1 activity and attenuated NO production (36). TAMs’ dysregulated arginine metabolism promotes tumor growth and progression by compromising the antitumoral immune response.

Tryptophan is another amino acid with an immunoregulatory function that was discovered roughly two decades ago. Indoleamine 2,3-Dioxygenase (IDO), which is increased in TAM, is the initial rate-limiting enzyme in the kynurenine pathway, depriving T cells of a critical nutrient and simultaneously driving regulatory Treg growth as an additional immunosuppressive strategy (39, 40). Central to the maintenance of peripheral tolerance at immune-privileged areas is the catabolism of the essential amino acid Trp into Kyn metabolites (39). The aryl hydrocarbon receptor (AHR) is a sensor of tryptophan metabolic products and a potent immune modulator. AHR activity was high in TAMs, and inflammatory phenotypes were produced in macrophages lacking AHR. In patients with pancreatic ductal adenocarcinoma (PDAC), elevated AHR expression is associated with rapid disease progression and mortality, as well as an immune-suppressive TAM phenotype (41), indicating that this regulatory axis is conserved in human illness.

### Lipid metabolism in TAMs

Increased FA metabolism is revealed in activated macrophages, which assists polarizing tissue macrophages to an M2 phenotype. Moreover, FA production in TAMs might be aided by the higher intracellular concentration of acetyl CoA (42). CD36, a specialized transporter, facilitates exogenous FA uptake from the environment. The accumulated level of FA uptaken by CD36 promotes TAMs fatty acid oxidation and oxidative phosphorylation to generate more energy for TAMs (43). TAM differentiation and inflammatory functions have been reported to be modulated by FA metabolism. FA-oxidation (FAO) acts as an alternative energy source, notably for M2 macrophages (44). FAO is essential for the protumoral function of macrophages and enhances the proliferation, migration, and invasion of hepatocellular carcinoma cells (HCC) (45). Upregulation of a critical FAO enzyme, carnitine palmitoyltransferase 1 (CPT1), protects cancer cells from glucose deprivation, while CPT1 knockdown makes cells more susceptible to therapy.

Conversely, overexpression of lipogenic enzymes such as acetyl-CoA carboxylase (ACC) and fatty acid synthase (FASN) is commonly seen in tumors and associated with poor prognosis (46). Genes associated in lipid metabolism, such as targets of the transcription factor sterol regulatory element-binding protein 1 (SREBP1), were also found to be enriched, implying that the transition from early to late tumor development may be linked to substantial metabolic reprogramming, as a prerequisite step for macrophage alternative activation (47). Through altering macrophage lipid metabolism, SREBP1 makes a significant contribution to the resolution phase of TLR4-induced gene activation (48). mTOR signaling stimulates fatty acid synthesis (FAS) through the induction of SREBP1 which in turn induced FASN and ACC (49).

Cholesterol is a crucial structural component of cellular membranes, and dysregulated cholesterol metabolism has an essential impact in a number of biological processes involved in the growth of tumors (50, 51). Cholesterol synthesis occurs in the cytosol, using acetyl CoA as its substrate (52). Extra cholesterol can be esterified and restrained in lipid droplets (LDs), or eliminated from the cell through the action of ABC transporters on the cell surface (53). It has been shown that ovarian cancer cells enhanced membrane cholesterol efflux and depletion of lipid rafts from macrophages, and increased cholesterol efflux facilitated IL-4-mediated remodeling, including the suppression of IFNγ-induced gene expression (54). Additionally, in a mouse model of bladder cancer, depletion of ATP-binding cassette transporter G1 (ABCG1), which is the cholesterol efflux transporter, suppresses tumor development through the buildup of cholesterol within macrophages (55), indicating that cholesterol accumulation in TAMs can abrogate their pro-tumor functions.

M2 macrophage phenotype is largely determined by the metabolism of arachidonic acid metabolism. Arachidonic acid decreased macrophage M2 polarization, but its derived metabolite prostaglandin E2 (PGE2)) facilitated it. Through inhibiting PPARγ, PGE2 resulted in alternative macrophage activation (56). Identification of PGE2 as a crucial factor in macrophage M2 polarization provides a metabolic interpretation for how tumors polarize infiltrating macrophages towards an immunosuppressive M2 type (57). Several macrophage-associated diseases, including malignancies, may be prevented by inhibiting the metabolic synthesis of PGE2 (56).

## TAMs as a target in cancer immunotherapy

### Reprogramming and repolarization of TAMs in cancer immunotherapy

Rapidly gaining importance in the treatment of the vast majority of tumors, cancer immunotherapy is a method that is becoming increasingly important. Immunotherapeutic techniques have been effective in treating a range of cancer subtypes and clinical hurdles still persist. Tremendous efforts have been devoted to targeting TAM for anticancer treatment, including diminishing, halting, or remodeling immune-suppressive M2-like macrophages (58). Current macrophage-based immunotherapeutic strategies are highly dependent on TAMs, which are polarized towards a pro-tumoral and immunosuppressive (M2) phenotype, and exhibit tumor-associated antigen specificity.

One of the most well-characterized techniques includes inhibiting colony-stimulating factor 1 (CSF-1) or its receptor, CSF1R, to deplete and/or decrease pro-tumor macrophages, resulting in CSF1R-dependent macrophage infiltration, thereby boosting immune-suppressing TMEs (59). CSF-1 is essential for the differentiation and development of macrophages. Studies have demonstrated that CSF1R mRNA expression is confined to myeloid cells. CSF1R and its ligands, as well as CSF1, and IL-34, play important roles in the regulation of Macrophage proliferation, differentiation, and survival (60). In conjunction with paclitaxel, blocking macrophage recruitment with CSF1R-signaling antagonists increased the longevity of mice with mammary tumors by decreasing primary tumor development and lowering pulmonary metastasis (59). It has been demonstrated that a potent and highly selective small molecule CSF-1R inhibitor, BLZ945, inhibits early gliomagenesis by downregulating markers of M2-like macrophage polarization/alternative activation and adopting a profound phagocytic phenotype (61). According to the findings of clinical trials, CSF1 and CSF1R inhibitors are typically well tolerated and show modest effectiveness (59, 61, 62).

Nearly a decade ago, targeting TME immunosuppressive components, including TAMs, showed promise in enhancing both immune checkpoint inhibitors (ICI) and Adoptive Cell Therapy (ACT). ICI is one of the cancer immunotherapy approaches with the highest promise. However, the majority of patients do not respond to ICI therapy. Targeting aspects of TME to overcome tumor resistance has shown promise in enhancing ICI therapy. The first macrophage-targeted checkpoint is CD47/SIRPα axis (63). CD47 is an essential tumor antigen for the initiation and progression of several cancer types (64). The interaction of Cluster of Differentiation 47 (CD47) with Signal regulatory protein alpha (SIRPα) prevents phagocytosis by triggering a “do not eat me” signal to the macrophages (64). The therapeutic action of anti-CD47 antibody alone or in combination with rituximab is predominantly mediated by macrophage phagocytosis, and macrophage depletion abolished the synergistic impact of anti-CD47 antibody in conjunction with rituximab, emphasizing the significance of macrophages as effectors of anti-CD47 antibody treatment in human non-Hodgkin lymphoma (65). Multiple therapeutics, including conventional antibodies, recombinant polypeptides, and bispecific molecules, which target the CD47-SIRPα axis, are undergoing preclinical and clinical settings (63, 65, 66). It has been documented that the Programmed death-1(PD-1) signal pathway modulates the phagocytic capability of TAMs and functions as the “do not eat me” signal (67). The function of PD-1 blockade on TAMs cannot be neglected, which may aid the search for new therapeutic strategies, which indicates broad and diverse possibilities for improving antitumor immunity with the potential to generate durable clinical responses, but still requires further investigations (68). Aside from tumor cell-target therapeutics like anti-CD47 antibodies and PD-1 blockade, other macrophage-related checkpoints are also identified, such as CD40 agonists, B7-H4 (aka B7x, B7S1 or VTCN1) and V-domain Ig- containing suppressor of T cell activation (VISTA, aka PD-1H, DD1α). CD40, a member of the family of tumor necrosis factor (TNF) receptors, is displayed by tumor cells and antigen-presenting cells (APCs), including macrophages. CD40-activated macrophages quickly invaded tumors, were tumoricidal, and aided the removal of tumor stroma. Combing a CD40 agonist with the chemotherapy drug gemcitabine in patients with surgically incurable pancreatic ductal adenocarcinoma led to tumor regressions in some individuals (69). Additional immune checkpoint ligands possibly represented by TAMs are B7-H4 and VISTA, which contribute to macrophages’ immunosuppressive capacity and suppression of T-cell activation (70).

The major histocompatibility complex class I (MHC-I), which is produced by tumor cells, also suppresses macrophage-mediated phagocytosis. It has been proven that tumor cells that express MHC I component β2-microglobulin (β2M), are straightly protected against phagocytosis. In addition, the researchers further showed that this protection was carried out by leukocyte immunoglobulin-like receptor subfamily B member 1(LILRB1), whose expression was amplified on the surface of TAMs (71). Despite promoting macrophage-mediated phagocytosis, MHC-I or LILRB1 blockage does not extensively suppress tumor growth in immunocompetent mice, highlighting some of the limits of this treatment (72). CD24, also known as small cell lung cancer cluster-4 antigen or the heat-stable antigen, is especially upregulated in tumor cells (67). CD24 mAb was found to increase macrophage phagocytosis. CD24/SIGLEC-10 axis inhibition showed an improvement in phagocytosis in MCL (73). The pro-phagocytic effects of CD24/SIGLEC-10 axis inhibition are much greater than those of CD47 blockade treatment (74), indicating that CD24 blockade is a promising immunotherapy strategy.

The immunosuppression of the TME is reversed by the polarizing tumor-enhancing M2 macrophages to anticancer M1 macrophages. It has recently come to light that reprogramming M2 macrophages into M1 macrophages may be a viable option for cancer immunotherapy (9). The following are involved in these reprogramming mechanisms: Poly (L-glutamic acid)-combretastatin A4 conjugate (PLG-CA4) (75), anti-macrophage receptors with collagenous structure (anti-MARCO) therapy (76), toll-like receptor (TLR) agonists (77), mucin domain containing 4 (Tim-4) blockades (78) and so on. PLG-CA4 is a member of a unique class of Vascular disrupting agents (VDAs) with promising cancer therapy potential. Synergizing with Phosphoinositide 3-kinase gamma (PI3Kγ) inhibitor, PLG-CA4 diminishes tumor metastasis in metastatic breast cancer by increasing the polarization of tumor-associated macrophages (TAMs) toward the M2-like phenotype (75). MARCO expression was found to be associated with the expression of M2 markers defined as being expressed by macrophages that promote tumor growth. An anti-MARCO monoclonal antibody has been shown to boost tumor immunogenicity in melanoma models, reprogram TAM populations to a pro-inflammatory phenotype, and induce antitumor action in breast and colon carcinoma in preclinical studies (76). The activation of TLR shifts macrophage polarization toward a more pro-inflammatory phenotype (77). The activities of different TLR ligands during TAM differentiation into tumor-killing macrophages have been studied in various cancer models (77). The M1 phenotype of activated macrophages is induced by TLR agonists, producing promising preclinical therapeutic effects against hematological cancers. In hematopoietic organs like bone marrow, spleen, lymph node, and fetal liver, Tim-4 is extensively expressed on the surface of macrophages. Recent studies have shown that suppressing Tim-4, which inhibits tumor progression and metastasis (79), increases the antitumor efficacy of anti-PD-1 treatment in the preclinical test, leading to its endorsement as a TAM target (78). To date, the mTOR pathway has been identified as a critical regulator of TAM differentiation throughout the process of angiogenic proliferation (80). Metformin shifts TAM polarization from the pro-tumor M2 to the antitumor M1 phenotype, lowers macrophage infiltration in tumors, and inhibits tumor growth and angiogenesis (81, 82).

Inhibiting class IIa histone deacetylases (HDACs) is a promising strategy to harness macrophages’ antitumor potential through epigenetic regulation. Macrophage transcription could be modified by TMP 195, a selective class IIa HDAC inhibitor. In preclinical models of breast, TMP195 inhibited macrophage-mediated tumor growth (83). The findings of this study have important clinical applications for TAM reprogramming in the future. When combined with standard chemotherapy (carboplatin or paclitaxel) or anti-PD1 antibodies, TMP195 dramatically reduced tumor size and enhanced T cell action compared to monotherapy (83). The efficacy of TAMs-related immunotherapy has been demonstrated in both preclinical and clinical settings, but there are still many challenges to be done to fully understand and exploit this crucial and promising tool in the battle against cancer. In 2020, a novel cellular therapy, Chimeric Antigen Receptor-macrophages (CAR-M), has been first established (84). CAR-Ms released pro-inflammatory cytokines and chemokines, convert bystander M2 macrophages to M1, increased antigen presentation machinery, attracted and presented antigen to T cells, and resisted the effects of immunosuppressive cytokines, as shown by the characterization of CAR-M activity (84). A variety of CAR-Ms has been assessed for the treatment of hematological malignancies and solid tumors in both preclinical studies and clinical studies (85, 86). Nevertheless, future studies should focus on the interplay between inhibiting “do not eat me” and activating “eat me” signal pathways as a means of antitumor treatment (72). Hence, TAM-targeted therapy enhances the efficacy of immune checkpoint blockade therapy and serves as a complementing component of cancer immunotherapy.

### Recruitment inhibition of TAMs in cancer immunotherapy

Another TAMs-targeting strategy for eliminating TAMs from TME is to prevent TAMs accumulation. TAMs are recruited by molecules such as TGF-β, macrophage CSF-1, chemokines such as CCL2, cytokines such as IL-4 and IL-1, immune complexes identified by receptors against the Fc component of immunoglobulin G (FcγR), and complement (6). CCL2 has been associated with a variety of tumor-promoting processes, such as the recruitment of TAMs, and the promotion of tumor cell invasiveness. There is great potential for a TAM-targeted treatment that interferes with CCL2/CCR2 signaling because of its essential regulatory role in circulatory monocytes and their infiltration into the TME (87). Genetic silencing and administration of a CCL2 neutralizing antibody or CCR2 antagonist inhibited the recruitment of circulatory monocytes, hence lowering the quantity of TAMs, and suppressing the secretion function of M2-like TAMs (88–90). Hypoxia-induced activation of stromal cell-derived factor 1 alpha (SDF-1α/CXCL12) also plays a role in the recruitment of the suppressive M2 macrophages (91). The CeXeC motif chemokine receptor 4 (CXCR4) antagonist AMD3100 blocked the polarization toward regional immunosuppression and paved the way for anti-PD-1 antibody treatment in a sorafenib-resistance HCC model (92). Additionally, PI3Kγ, the only member of the class-1B family, supports an increasing viable activation (M2) immunosuppressive condition in tumor-associated macrophages (93). Decreased accumulation of macrophages in the glioblastoma microenvironment and breast cancer has been recapitulated by pharmacologic inhibition or genetic inactivation of PI3Kγ, suggesting PI3Kγ inhibition as a promising strategy for cancer therapy (75, 94). Together, inhibitors of PI3Kγ have a synergistic impact with checkpoint inhibitor therapy to reduce tumor growth and enhance the prognosis of animal models with tumors (95).

### TAMs elimination in cancer immunotherapy

TAMs clearance is a potential strategy for mitigating the adverse effects of TAMs during immunotherapy. In terms of their molecular mechanism of action and chemical composition, Bisphosphonates can be categorized into two broad classes: non-nitrogen-containing and nitrogen-containing. Clodronate is a member of the non-nitrogen bisphosphonates family, while Zoledronate belongs to the family of nitrogen-containing bisphosphonates (96, 97). Clodronate-loaded liposomes (clodrolip) were commonly utilized to eliminate liver macrophages. Preclinical models showed reduced tumor growth and improved survival after the administration of clodrolip-depleted TAMs (98, 99). Diminishing angiogenesis and tumor growth by depleting TAMs with approaches including CSF1 inactivation, CSF1 receptor (CSF1R) antibodies, and clodronate liposomes has been shown in a variety of tumor types (100, 101). Depletion of macrophages by clodrolip or Zoledronate acid (ZA) in conjunction with sorafenib dramatically decreased tumor development, tumor angiogenesis, and lung metastasis in mice when compared to sorafenib alone (99). ZA blocks the formation of the mevalonate pathway downstream metabolite geranylgeranyl diphosphate by inhibiting farnesyl diphosphate (FPP) synthase (96). Inhibiting the FPP enzyme hinders the biosynthesis of isoprenoid lipids, which are required for the prenylation of small GTPase signaling proteins (96). Because of its ability to induce macrophage apoptosis, trabectedin has been approved for use as a second-line antineoplastic drug for the treatment of advanced soft tissue sarcoma and ovarian cancers (102). Trabectedin has been shown to be an excellent predictor of antitumor activity in breast cancer due to its capacity to deplete pro-tumoral TAMs and down-regulate inflammatory cytokines and angiogenic factors (103). A significant reduction in TAM density has been observed in tumor biopsy specimens of patients with soft tissue sarcoma after trabectedin treatment (104). The use of trabectedin able to abolish TAMs represents a potential target in cancer immunotherapy.

## Metabolic modulation of TAMs in cancer immunotherapy

Metabolic reprogramming is a hallmark of malignant tumors. Macrophages are prompted to adopt an immunosuppressive phenotype when cancer cells secrete lactate, glutamine, succinate, and a-ketoglutarate (a-KG). Furthermore, metabolic reprogramming of TAMs allows for the buildup of T cell receptor-engineered T cells, which in turn inhibits tumor progression. The anti-cancer efficacy of immune checkpoint blockade therapy may be enhanced by combination with TAMs-targeted metabolic modulation in a comprehensive approach. Metabolic modulation strategies that target TAMs in cancer immunotherapy are summarized in **Table 1**.

**Table 1:** Selected agents targeting TAMs metabolism.

Targeted TAMs metabolism	Drug	Targeted Agent	Metabolic modulation	Functions on TAMs	Ref.
Glucose metabolism	2-DG	Hexokinase 2	Aerobic glycolysis inhibition	Macrophage phagocytosis, Repolarization of TAMs	(31, 105, 106)
Glucose metabolism	Compound 1	Lactate dehydrogenase	Decreased lactate production Interfering TME acidity	Increased M1/M2 ratio in TME	(107)
Glucose metabolism	CHC	MCT	sustaining high glycolysis	Repolarization of TAMs	(26)
Glucose metabolism; Lipid metabolism	metformin	AMPK mTOR	increased lipid breakdown, Inhibition of glycolysis	Decreased M2 polarization of macrophages; macrophage infiltration	(25, 81, 82)
Glucose metabolism; Lipid metabolism	Olaparib, Niraparib, Talazoparib	SREBP PARP	Switch from glycolysis to lipid metabolism, metabolic reprogramming	modulate macrophage state, phenotype, function, and metabolism.	(108)
Glucose metabolism	Shikonin, HA344,	PKM2	Inhibition of glycolysis	Suppresses cancer cell proliferation and overcomes, chemotherapeutic drug-mediated resistance	(19, 109, 110)
Glucose metabolism; Lipid metabolism	HOE642	NHE1	Increases glucose uptake with metabolic shifts towards the OXPHOS metabolism	TAMs polarization and metabolism	(111, 112)
Glucose metabolism; Lipid metabolism	DCA AlbA	PDK	Metabolic shift from glycolysis to OXPHOS	Repolarization of TAMs	(113, 114)
Amino acid metabolism	AHRi1, CH-223191	AHR	Tryptophan metabolism	Repolarization of TAMs	(39)
Amino acid metabolism	JHU083	GLS1/2 glutamine	Blocking of glutamine metabolism	Increased M1/M2 ratio in TME, Increased antitumor inflammatory TAMs	(115, 116)
Amino acid metabolism	Methionine Sulfoximine	Glutamine Synthetase	Inhibition of glutamine metabolism, impaired glutamine usage by TAMs	Repolarization of TAMs	(117)
Amino acid metabolism	Glufosinate	Glutamine Synthetase	Inhibition of glutamine metabolism	Repolarization of TAMs	(118)
Amino acid metabolism	L-Norvaline, CB-1158	Arginase	Inhibition of arginine synthesis, Decreasing arginine levels	Increased anti-tumoral T cell response in TME, Repolarization of TAMs	(36)
Lipid metabolism	Fatostatin	SREBP	Inhibition of FA metabolism	M2-like TAM polarization	(119)
Lipid metabolism	Etomoxir	CPT1	Impaired lipid oxidation	Decreased M2 activity, TAM infiltration	(120, 121)
Lipid metabolism	C75 TVB-2640	FASN	Inhibited lipid droplet formation	Increased M1/M2 ratio in TME	(122)
Lipid metabolism	NCX-4016, YVAD, VAD	Caspase-1/PPARγ	Inhibition of lipid accumulation	TAMs repolarization and differentiation	(123)
Lipid metabolism	ATR-101	ABCA1/ABCG1	Inhibition of cholesterol efflux	Reprogramming TAMs	(124)

### TAMs glucose metabolism in cancer immunotherapy

Since glycolysis is crucial for the tumor-promoting actions of TAMs, blocking the glycolysis pathway has been investigated as a means to promote the repolarization of TAMs. Glucose is critical for the proliferation and activation of M1 macrophages. The majority of the studies focusing on glycolysis to reverse macrophage polarization have utilized glycolytic inhibitors, such as 2-deoxy-d-glucose (2-DG) (105). Disruption of this pro-metastatic phenotype by 2-DG, a competitive inhibitor of Hexokinase II (HK2), resulted in a reversal of the increases in angiogenesis, extravasation, and EMT that were facilitated by TAMs (31). Similarly, 2-DG also inhibits the phagocytosis activity of elicited macrophages (106). The effectiveness of 2-DG alone and in combination with docetaxel in treating advanced solid tumors was evaluated in a completed phase I clinical trial (125). The cellular uptake of lactate produced by tumor cells is mediated by MCTs in TAMs. A-cyano-4-hydroxycinnamate (CHC), a monocarboxylate channel transporter (MCT) inhibitor significantly abrogated ARG1 expression, suggesting that MCTs promote polarization towards the M2-like phenotype and help sustaining high glycolysis (26). Metformin, an antidiabetic agent, was shown to re-modulate the TME in preclinical models, decreasing TAMs’ density while increasing their phagocytic function (126). Decreasing lactate synthesis in the TME was achieved by lactate dehydrogenase inhibitor (Compound 1), which in turn increased the M1/M2 ratio (107). Treated with olaparib, a PAPR inhibitor, the Glycolytic capacity of TAMs was markedly decreased (108). Strikingly, CSF1R blockade also causes a profound metabolic rewiring, with the restoration of glycolysis to favor the maintenance of M1-like TAMs (127). An important enzyme in the final step of glycolysis, pyruvate kinase M2 (PKM2), promotes aerobic glycolysis in cancer cells during tumor progression and serves as a regulatory site of numerous signaling pathways (109). As a PKM2 inhibitor, Shikonin has been shown to reduce cancer cell proliferation and reverse chemotherapeutic drug-mediated resistance (19, 109). Additionally, HA344, which is thought to be a potential application for overcoming cancer resistance, hinders the terminal and rate-limiting phases of glycolysis by covalently binding to PKM2 and blocking the activity of inosine monophosphate dehydrogenase (110). The PDK inhibitor, Dichloroacetate (DCA), causes a metabolic switch from glycolysis to OXPHOS in M1 macrophage by activating pyruvate dehydrogenase complex (PDC) in M1 and M2. In addition, DCA significantly amplifies M1 and M2 reactive oxygen species (ROS) production (113). Albiziabioside A (AlbA), a natural oleanane triterpenoid saponin, which is bound to DCA, reshapes the tumor immunosuppression microenvironment by eradicating M2-TAMs, hence preventing both primary and distant tumor progression (114). The Na/H exchanger (NHE1) governs the cellular pH hemostasis in all types of cells (111). Inhibiting NHE1 in TAMs, either on its own or in combination with temozolomide, switches TAMs metabolism from glycolysis to OXPHOS and enhances the antitumor function of TAMs (21, 111). Additionally, the NHE1blocker HOE642 promotes antitumor macrophage activity by increasing TAMs’ glucose uptake and shifting their metabolism toward OXPHOS metabolism (111). Moreover, a new technique for enhancing tumor immunogenicity for cancer immunotherapy was presented by combining anti-PD-1 antibody treatment with HOE642. This resulted in a considerable extension of median survival in the mouse glioma model (112).

### Amino acid metabolism of TAMs in cancer immunotherapy

One of the immunosuppressive mechanisms facilitated by both TAMs and MDSCs is AA metabolism, notably tryptophan metabolism via the enzyme IDO. One of the mechanisms underlying the resistance to immune checkpoint inhibitors has been identified as IDO. IDO-1 inhibitors may be of special interest in the treatment of sarcoma, either alone or in conjunction with lymphocyte-targeting therapies (66). The use of AHR pathway inhibitors in IDO/TDO expressing malignancies represents a tailored strategy to immunotherapy, especially when used in conjunction with immune checkpoint inhibitors, by circumventing the limitations of single IDO or TDO targeting drugs (39). The use of small molecule antagonists to inhibit AHR signaling has been demonstrated to prevent nuclear translocation of AHR, increase the production of IFN-γ, TNFɑ, and IL-2, and decrease the number of TAMs (128). Combinations of IDO inhibitors with immune checkpoint inhibitors are currently being studied. As a prodrug blocking glutamine metabolism, JHU083 has had effects not only on cancer cells but also acted on the immune component of the TME. It boosted antitumor immunity by increasing the production of antitumor inflammatory TAMs and reduced the metastatic potential of tumors (115, 116). In mice with Lewis lung carcinoma, tumor growth was suppressed by using a GS-blocking drug, methionine sulfoximine (MSO) to convert pro-tumoral M2 macrophages into antitumoral M1 macrophages (117). Glufosinate, a glutamate analogue, is a non-proteinogenic amino acid that reduces GS by competing with glutamate for binding in the active site (129). At both the primary tumor and the metastatic site, glufosinate rewires macrophages towards an M1-like phenotype, countering immunosuppression and stimulating vascular sprouting, however, TAM elimination by anti-CSF1R substantially neutralized glufosinate effects on metastasis (118). The possibility of targeting metabolic checkpoints in macrophages to treat cancer metastasis is highlighted by the identification of GS as a druggable enzyme that rewires macrophage activity. By activating inducible NO synthase (iNOS) and arginase, macrophages can convert L-arginine into nitric oxide (NO) and polyamines. L-norvaline, an arginase inhibitor, can reduce tumor cell proliferation brought on by the overexpression of arginase in macrophages, which increased the synthesis of L-ornithine and putrescine production (36).

### Inhibition of lipid metabolism in TAMs in cancer immunotherapy

The lipid-dependent macrophages in the TME contribute to the immunosuppressive environment, and blocking FA metabolism in TAMs has been proposed as a way to boost the antitumor effect of cancer treatment. Inhibitors of FA synthesis (FAS) and SREBP1 inhibitors, such as Fatostatin, have been demonstrated to improve the efficacy of checkpoint blockade therapy (119). There has been extensive research on the enzyme inhibitors of enzymes of lipid metabolism (FASN, ACC, CPT1). As a CPT1 inhibitor, etomoxir abolished the transition of macrophages toward the immunosuppressive M2 phenotype and promotes the pro-inflammatory M1 phenotype (120), effectively reversing the accumulation of TAMs in HCC tissues (121). By blocking FASN, C75 induced M1 phenotype via MEK1/2 axis in the Raw264.7 macrophage cell line and suppressed lipid droplet production (122). Caspase-1 cleaves peroxisome proliferator-activated receptor gamma (PPARγ), which limits FA oxidation, thereby accumulating lipid droplets and promoting tumor-associated macrophage differentiation. Treatment with caspase-1 inhibitors (YVAD, VADs, and NCX-4016) suppressed lipid accumulation and primary tumor growth for targeting the caspase-1/PPARγ/MCAD pathway in TAM differentiation (123). Palmitate treatment decreased phagocytosis, inhibited the production of pro-inflammatory cytokines, and led to M2 polarization (130). Olaparib, a PARP inhibitor, alters macrophage metabolism, particularly in lipid metabolic processes and in β-oxidation of FA analyzed by RNA-seq and proteomic analyses (108), and the researchers further speculate that the SREBP1 pathway is a main regulator of the olaparib-induced macrophage phenotype.

Cholesterol is crucial to tumors since it is used extensively by cancer cells for their proliferation (131). As a novel therapeutic strategy, targeting cholesterol metabolism is gaining growing interest. Therefore, efforts to either reduce cholesterol synthesis or restrict cholesterol uptake have been proposed as viable anticancer treatments (124, 132). Reverse cholesterol efflux in macrophages through membrane cholesterol efflux transporters, including ATP binding cassette transporter A1(ABCA1) and ABCG1 may be a novel target to supersede the pro-tumor functions of TAMs while keeping potentially advantageous antitumor actions in response to treatment (54). ATR-101, an inhibitor of the cholesterol efflux transporters ABCA1 and ABCG1, has acted as a reprogramming agent for TAMs. It is noteworthy that ATR-101 therapy also partially reversed the effects of tumor-cell conditioned medium on the expression of HIF1A, VEGFA, and CXCL8 in TAM-like cells, but M0 macrophages were unaffected (124). Because of its potential to block acyl-coenzyme A: cholesterol O-acyltransferase 1 (ACAT1), an enzyme that catalyzes the esterification of intracellular FC, ATR-101 has been explored as a candidate for the treatment of adrenocortical carcinoma (133).

### Metabolic alteration when crosstalk with T cells

Critical to the TME and the advancement of the tumor, Tregs have been shown to directly stimulate the differentiation of monocytes into immunosuppressive TAMs. When activated, SREBP1 inhibits FA synthesis in immunosuppressive (M2-like) TAMs, however, Treg cells restrict IFN-γ release from CD8+ T cells, allowing their cells to produce FAs normally. Fatostatin, an SREBP1 inhibitor, boosts the effectiveness of checkpoint blockade by reinvigorating CD8+ T cells, and more crucially, stimulating their inherent ability to repress M2-like TAMs, thereby fostering a strong antitumor immune response (119). Fatostatin and anti-PD-1 combination slowed tumor development and prolonged survival, while either Fatostatin or anti-PD-1 treatment alone provided a limited therapeutic advantage (119). Thus, Tregs indirectly but selectively maintained metabolic fitness, mitochondrial integrity, and survival in M2-like TAMs. Driven by the SREBP1 pathway, Poly (ADP-ribose) polymerase (PARP) inhibitors improve macrophages’ anti- and pro-tumor properties through glucose and lipid metabolic reprogramming. Combining PARP inhibitor therapy with CSF1R-blocking antibodies greatly improved innate and adaptive antitumor immunity and prolonged survival in mice with BRCA-deficient tumors *in vivo*, with CD8+ T cells serving as the mediators (108). Tregs and TAMs preserve immunosuppressive pro-tumoral functions by activating the AHR pathway in the presence of high IDO or TDO expression (39). AHR inhibition reverses IDO-Kyn-AHR-mediated immunosuppression, which is dependent on an interplay between Tregs and TAMs, and it slows development in IDO/TDO overexpressing tumors; its efficacy is enhanced when combined with PD-1 blockade (39).

## Conclusions and perspectives

**Figure 2:**
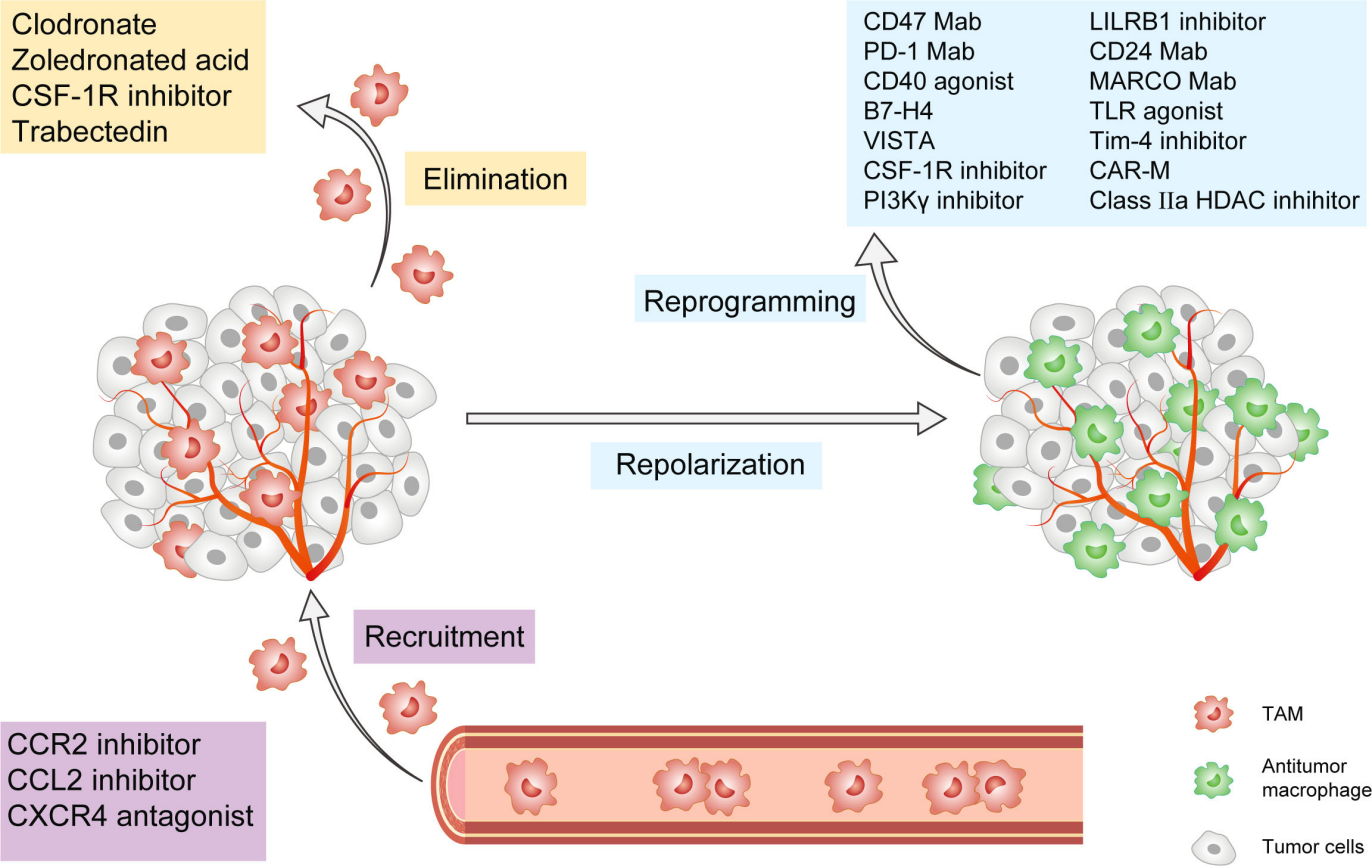
Cancer Immunotherapy targeting tumor-associated macrophages. This figure presents the popular strategies targeting TAMs in preclinical or clinical studies. These strategies are classified into three groups: 1) Inhibition of TAMs recruitment, including CCL2/CCR2 axis, CXCR4, and PI3Kγ pathway; 2) Elimination of TAMs already present in tumor tissue, including CSF-1/CSF-1R axis, trabectedin, and bisphosphonates; 3) Reprogramming and Repolarization of TAMs, including CD47, PD-1, CD24, and MARCO mAb, as well as CD40 agonists, B7-H4, VISTA, CSF-1R inhibitor, PLG-CA4, LILRB1 inhibitor, TLR agonist, Tim-4 agonist, Class IIa HDACs inhibitors and CAR-M. TAM, tumor-associated macrophage; CCL2, CeC motif chemokine ligand 2; CCR2, CeC motif chemokine receptor 2; CSF-1R, colony-stimulating factor-1 receptor; CXCR4, CeXeC motif chemokine receptor 4; PI3Kγ, phosphoinositide 3-kinase-γ; PD-1, CD24, and MARCO, macrophage receptors with collagenous structure; PLG-CA4, Poly(L-glutamic acid)-combretastatin A4 conjugate, LILRB1, Leukocyte immunoglobulin-like receptor subfamily B member 1; TLR, toll-like receptor; Tim-4, mucin domain containing 4; HDACs, histone deacetylases; CAR-M, Chimeric Antigen Receptor-macrophages.

TAMs govern tumor progression and metastasis by influencing the TME and immune ecology. Metabolic targeted therapy can help shape the functional programs activated by TAMs in the tumor microenvironment in several ways, including by interfering with TAMs recruitment, promoting TAMs depletion, and reprogramming pro-tumoral TAMs towards an antitumoral phenotype (**Figure 2**). As a result, they can be taken into account in the perspective of novel preclinical and clinical approaches (**Table 1**). Current preclinical and clinical data suggest that anti-TAM treatment should be used with conventional chemotherapy in order to suppress tumor progression and regulate TME, hence achieving more pronounced clinical effects on patients. Targeting metabolic activities essential for the formation of protumoral macrophage phenotypes is a safe and effective technique for inhibiting TAMs’ immunosuppressive activity.

However, current TAM-related therapeutic strategies focus mostly on exhausting TAMs in TME, whereas anti-metabolizing drugs tend to inhibit tumor cell metabolism rather than TAMs. Therefore, a better characterization of the mechanism underlying the intrinsic metabolic signals associated with TAMs activation through cross-talk with cancer cell-derived metabolites is required for further investigation. More efforts are needed to further understand metabolic alteration and its modulation of TAMs in cancer in order to discover more precise targets and hallmarks that will aid in the development of cancer immunotherapy that is more effective and safer.

## Author contributions

LX, QW, and HP performed the literature search and screening. LX drafted the manuscript. HP reviewed and revised the draft. All authors contributed to the article and approved the submitted version.
